# Draft genome sequence of *Kluyveromyces marxianus* MCHA

**DOI:** 10.1128/mra.01266-25

**Published:** 2026-04-20

**Authors:** Gabriel Ibarra-Rivera, Luis Alfonso Muñoz-Miranda, Alejandro Pereira-Santana, Melchor Arellano-Plaza, Anne Gschaedler-Mathis, Manuel R. Kirchmayr, Luis Joel Figueroa-Yañez

**Affiliations:** 1 Industrial Biotechnology Unit, Center for Research and Assistance in Technology and Design of the State of Jalisco, A.C. (CIATEJ), Guadalajara, Jalisco, Mexico; 2 Center for Research on Infectious Diseases, Department of Microbiology and Pathology, University Center for Health Sciences, University of Guadalajara, Guadalajara, México; 3 SECIHTI-Research and Assistance Center in Technology and Design of the State of Jalisco, Parque Científico Tecnológico de Yucatán, Mérida, A.C. (CIATEJ) 98806, Mérida, Yucatán, Mexico; Rochester Institute of Technology 6925, Rochester, New York, USA

**Keywords:** *Kluyveromyces marxianus*, genome sequence

## Abstract

The complete genome sequence of *Kluyveromyces marxianus* strain MCHA was obtained using Illumina and Oxford Nanopore sequencing technologies. The genome comprises nine scaffolds, corresponding to the eight nuclear chromosomes and the mitochondrial genome.

## ANNOUNCEMENT

*Kluyveromyces marxianus* is an emerging nonconventional food-grade yeast isolated from diverse environments, such as fermented products, industrial waste, and plants (1). In fermented foods and beverages, it contributes to aroma formation through the production of volatile organic compounds (VOCs) (2). Its rapid growth rate, thermotolerance, and metabolic versatility have positioned *K. marxianus* as a promising organism for biotechnological applications (3). We report the draft genome sequence of *K. marxianus* strain MCHA isolated from *Agave cupreata* juice fermentations in a mezcal factory located in Piedras de Lumbre, Morelia, Michoacán, Mexico. The strain was obtained by serial dilution plating on YPD medium (10 g L⁻¹ yeast extract, 20 g L⁻¹ peptone, and 20 g L⁻¹ glucose; pH 6.0) and incubated at 30°C for 24–48 h with shaking at 200 rpm. Taxonomic identification was confirmed by matrix-assisted laser desorption/ionization time-of-flight (MALDI-TOF) mass spectrometry.

Genomic DNA was extracted from fresh yeast cultures grown in YPD medium at an optical density of 1.5 using the Wizard Genome DNA Purification Kit (Promega, USA) following the manufacturer’s instructions. DNA was sent to Novogene Co., Ltd. (Beijing, China) for library preparation and sequencing. Illumina libraries were prepared using the TruSeq DNA PCR-Free Library Preparation Kit (Illumina, USA) according to the manufacturer’s instructions. Sequencing was performed on an Illumina HiSeq 2000 platform (2 × 100 bp), generating 6,309,056 paired-end reads. Read quality was assessed using FastQC v0.11.9 (4), and adapter sequences and low-quality bases were removed using Trimmomatic v0.39 (5).

For Oxford Nanopore sequencing, genomic DNA was processed without prior mechanical shearing. Libraries were prepared using the Rapid Barcoding Kit (SQK-RBK004; Oxford Nanopore Technologies, UK) according to the manufacturer’s protocol, without additional size selection. Sequencing was performed on a MinION Mk1B device using a FLO-MIN106 (R9.4.1) flow cell. Basecalling was performed using Guppy v6.3.8 (Oxford Nanopore Technologies) with GPU support, and adapter trimming and demultiplexing were performed with Guppy’s internal scripts. Low-quality and contaminant reads were filtered using NanoFilt v2.8.0 (6) and Filtlong v0.2.1 (7).

High-quality long reads (*n* = 67,953; total bases = 252.5 Mb) were assembled using Flye v2.9.1 (8) with the --nano-raw option and an estimated genome size of 11 Mb. The assembly was polished using Minimap2 v2.24 (9) and four rounds of Racon v1.5.0 (10), followed by Medaka v1.6.0 (11). A second round of polishing was performed using Illumina data with Pilon v1.23 (*--fix all option)* (12). Alignment of the Illumina reads to the assembly was carried out using Bowtie 2 (13).

The final assembly was scaffolded with RagTag (14) using the reference genome of *K. marxianus* DMKU3-1042 (GCA_001417885.1). Assembly quality was evaluated with QUAST v4.1 (15), and completeness was assessed using BUSCO v5.8.2 (16) with the Saccharomyces database. Gene prediction and functional annotation were performed with Funannotate v1.8.14 (17) using *Kluyveromyces lactis* as the training species. The final genome assembly consisted of nine scaffolds with high completeness based on BUSCO analysis. Default parameters were used for all software unless otherwise specified. Detailed sequencing, assembly, and annotation metrics are summarized in Table 1.

**TABLE 1:** Genome sequencing statistics.

Parameter	Value
Oxford Nanopore sequencing	
Read number	67,953
Total bases (nt)	252,503,966
Illumina sequencing	
Read number	6,309,056
Total bases (nt)	946,358,400
Genome assembly	
Total length	10,881,469 pb
Number of scaffolds	9
Longest scaffold	1,734,893 pb
N50	1,410,736 pb
GC content (%)	40.03
Genome coverage	21.35×
Protein-coding genes	4,787
tRNA genes	187
Genome assembly assessment	
Complete BUSCOs	98.3%
Fragmented BUSCOs	0.4%
Missing BUSCOs	1.3%

## Data Availability

This Whole Genome Shotgun project has been deposited at DDBJ/ENA/GenBank under accession JBUZOE000000000. The version described in this paper is JBUZOE010000000. The raw sequencing reads are available at the NCBI Sequence Read Archive under accession numbers SRR34364681 and SRR34364682. The version described in this paper is the first version.
